# Heavy Metals in Surface Soils in the Upper Reaches of the Heihe River, Northeastern Tibetan Plateau, China

**DOI:** 10.3390/ijerph13030247

**Published:** 2016-02-23

**Authors:** Jianwei Bu, Ziyong Sun, Aiguo Zhou, Youning Xu, Rui Ma, Wenhao Wei, Meng Liu

**Affiliations:** 1 Geological Survey, China University of Geosciences, Wuhan 430074, China; jangous@163.com (J.B.); aiguozhou@cug.edu.cn (A.Z.); weiwenhao048@outlook.com (W.W.); lethelm@126.com (M.L.); 2 Laboratory of Basin Hydrology and Wetland Eco-restoration, China University of Geosciences, Wuhan 430074, China; rma@cug.edu.cn; 3 School of Environmental Studies, China University of Geosciences, Wuhan 430074, China; 4 Xi’an Center of Geological Survey, China Geological Survey, Xi’an 710054, China; ksdzhj@sohu.com; 5 Faculty of Mechanical & Electronic Information, China University of Geosciences, Wuhan 430074, China

**Keywords:** heavy metals, soil, Heihe River, Tibetan Plateau, multivariate statistics, risk assessment

## Abstract

The upper reaches of the Heihe River have been regarded as a hotspot for phytoecology, climate change, water resources and hydrology studies. Due to the cold-arid climate, high elevation, remote location and poor traffic conditions, few studies focused on heavy metal contamination of soils have been conducted or reported in this region. In the present study, an investigation was performed to provide information regarding the concentration levels, sources, spatial distributions, and environmental risks of heavy metals in this area for the first time. Fifty-six surface soil samples collected from the study area were analyzed for Cr, Mn, Ni, Cu, Zn, As, Cd and Pb concentrations, as well as TOC levels. Basic statistics, concentration comparisons, correlation coefficient analysis and multivariate analyses coupled with spatial distributions were utilized to delineate the features and the sources of different heavy metals. Risk assessments, including geoaccumulation index, enrichment factor and potential ecological risk index, were also performed. The results indicate that the concentrations of heavy metals have been increasing since the 1990s. The mean values of each metal are all above the average background values in the Qinghai Province, Tibet, China and the world, except for that of Cr. Of special note is the concentration of Cd, which is extremely elevated compared with all background values. The distinguished ore-forming conditions and well-preserved, widely distributed limestones likely contribute to the high Cd concentration. Heavy metals in surface soils in the study area are primarily inherited from parent materials. Nonetheless, anthropogenic activities may have accelerated the process of weathering. Cd presents a high background concentration level and poses a severe environmental risk throughout the whole region. Soils in Yinda, Reshui daban, Kekeli and Zamasheng in particular pose threats to the health of the local population, as well as that of livestock and wildlife.

## 1. Introduction

Soil is a means of transmittance for many pollutants to the atmosphere, groundwater and plants, as well as a potential sink for pollutants [1]. Heavy metals (HMs) are among the most significant soil pollutants and are particularly notorious due to their toxic effects, wide variety of sources, persistence and bio-accumulation [2,3,4]. HMs can enter the human body by ingestion, dermal contact and inhalation [5]. In general, HMs, such as chromium (Cr), manganese (Mn), nickel (Ni), copper (Cu), zinc (Zn), cadmium (Cd) and lead (Pb), are metals having densities greater than 5 g/cm^3^. Due to similarities in both chemical properties and environmental behaviors, the metalloid arsenic (As) is often grouped in the HM category [6]. HMs and associated compounds are naturally ubiquitous throughout soil environments [7]. They are introduced naturally through the weathering of parent materials, erosion, forest fires, volcanic eruptions and a variety of human activities, such as industry, mining, smelting, refining, electroplating, agriculture fertilization and drainage, traffic emissions and even domestic discharges [8,9,10,11,12].

Due to their potential toxicity, persistence, bio magnification and irreversibility, HMs have been listed as priority control pollutants by the United States Environmental Protection Agency (EPA) and have garnered attention in both developed and developing countries worldwide [13,14,15,16]. In recent decades, a large amount of soil pollution surveys on HMs have been conducted at different scales, and many of them have been reported in the scientific literature [6,17,18,19,20,21,22,23]. However, most of these studies have focused on cities (*i.e.*, industrial and residential areas), aquatic sediments (*i.e.*, rivers, lakes, bays and coasts), transportation areas (*i.e.*, railways and highways), non-urban mines and agricultural fields, where sites are easily accessible. As a result, in isolated regions, there is a pressing need to estimate local background concentrations and spatial distribution characteristics of HMs in soil, as well as the factors affecting their evolution [24].

The Tibetan Plateau and surrounding mountains are referred to as the “Third Pole of the World” due to their high altitude (over 4000 m), large territory (more than 3 million km^2^) and unique geographical position and climate [25,26]. The Tibetan Plateau is also called the “Water Tower of Asia” because it is the source region for over 10 of the largest rivers in Asia, including the Yangtze River, the Yellow River, the Brahmaputra River, the Ganges River and the Indus River, which provide water resources for approximately 40% of the World’s population [27]. Among these rivers, the Heihe River is the second largest inland river in China, originating in the Qilian Mountains (Qinghai Province in the northeast Tibetan Plateau) and flowing through an area of ~130,000 km^2^ before terminating in Juyanhai Lake (Inner Mongolia in the southwest Mongolian Plateau) [28].

The upper reaches of the Heihe River are regarded as a hotspot for the study of alpine phytoecology, regional/global climate change, water resources and ecological hydrology due to its varied landscape types, moderate river basin, fragile ecosystem, special monsoon circulation and complicated social-environmental issues [29]. In addition to having unique hydrology, biology, climate and landform, this area also has notable geological and metallogenic conditions. The upper reaches of the Heihe River contain 60% of the total annual water resources in the basin (1.6 billion cubic meters) and may be the key to solving water resource problems for the arid middle and lower reaches. The water resource dilemma is the core issue in maintaining and promoting the sustainable ecological, social and economic development of the entire Heihe River basin [30]. Thus, the soil and water quality in the upper reaches of the Heihe River are extremely important because they impact water usage and the environment downstream. Currently, human activities such as mineral resource exploitation, transportation and agriculture have become more frequent and have caused more contamination in the area. Although the area is drawing increasing attention from the government, populace and scholars, to our knowledge, almost no related studies on soil HMs have been conducted or reported until now.

The objectives of the present study were: (1) to determine the background concentrations of eight HMs (Cr, Mn, Ni, Cu, Zn, As, Cd and Pb) and their contamination levels; (2) to identify their main sources using multivariate statistics; (3) to illustrate their spatial distributions with GIS; and (4) to assess their environmental risks in the upper reaches of the Heihe River, northeastern Tibetan Plateau.

## 2. Materials and Methods

### 2.1. General Setting of the Study Area

**Figure 1:**
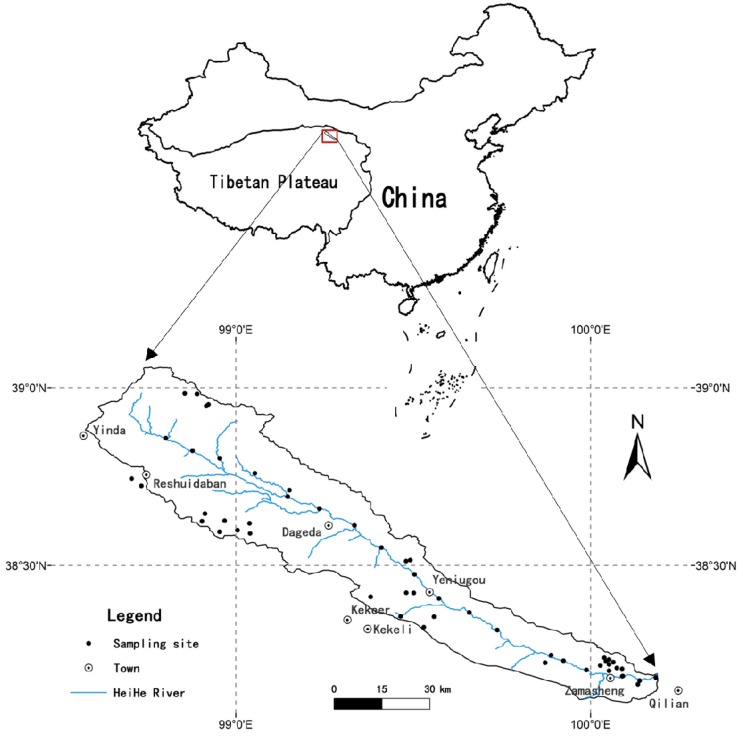
Map of the study area and surface soil sampling sites.

The upper reaches of the Heihe River are located in the Qilian Mountains of Qinghai Province and occupy an area of 3622 km^2^ (39°5′–38°9′N; 98°34′–100°11′E; Figure 1). This region extends as a narrow belt from the northwest to the southeast at the southern margin of the Qilian Mountains and the Gansu Corridor of the ancient Silk Road. The upper reaches of the Heihe River are characterized by an average elevation above 3200 m with various topographic features, including the famous Heihe River Grand Canyon. The climate is alpine continental, cold and arid, with a mean annual air temperature of 1 °C and an average annual precipitation of 420 mm. Due to the high altitude and low temperature, glaciers, permafrost, seasonal frozen soil and thermokarst ponds are extensively distributed. There are also multiple types of vegetation in the area, such as subtropical forest, temperate forest, shrub, grassland, meadow and prairie.

### 2.2. Soil Sampling

Fifty-six surface soil samples (0–20 cm in depth) were collected in the area during August 2013. To obtain representative samples, a series of standard soil sampling procedures were adopted [31,32,33]. Approximately 1 kg of fresh soil was collected using a clean plastic dustpan and brush and was stored in plastic bags at each site [34]. Prior to analysis, all samples were air dried at ~20 °C; sorted through a 2-mm plastic sieve to remove gravel-sized stones, large plant roots and other debris; ground; homogenized with an agate mortar; and passed through a 200-mesh sieve.

Sampling sites (Figure 1) were identified using a global positioning system (GPS). To acquire the background soils, all the samples were collected ≥100 m aside from any roads or cultivated fields to avoid the traffic influence and agricultural impact. The desolation, high altitude, harsh weather conditions and undeveloped infrastructure make a routine sampling campaign in the area very challenging. Thus, sampling sites in this study were somewhat unevenly distributed.

### 2.3. Chemical Analyses

The concentrations of eight HMs (Cr, Mn, Ni, Cu, Zn, As, Cd and Pb) considered of high environmental importance were measured. Soil total organic carbon (TOC) was also determined to better understand the spatial variations and its associations with different metals in the area [35].

A small portion (0.1 g) of milled soil was collected and placed in a polypropylene vessel and mixed with 2 mL of concentrated HNO_3_ and 1 mL of HClO_4_. The solution was heated on an open hot plate for approximately 4 h, or until white fumes were given off, and then the residue was re-dissolved in a plastic bottle with 2 mL of 4 mol·L^−1^ HCl and diluted to 10 mL with deionized water.

The concentrations of the eight HMs were measured by inductively coupled plasma mass spectrometry (ICP-MS, 7500a, Agilent, Santa Clara, CA, USA) in State Key Laboratory of Geological Processes and Mineral Resources, China University of Geosciences. In this study, ^115^In was used as an internal standard. The national standard reference samples GSS-1 and GSR-1 were used for quality control of the analyses. The measured values for reference samples were within the range of 87% to 102% of the certified values for all eight HMs, and the corresponding relative standard deviation values (analytical precision) were less than 5%. Total organic carbon (TOC) in soil (as a percent) was calculated from the difference between total carbon (TC) and inorganic carbon (IC), which were measured using a TOC analyzer (Vcph, Shimadzu, Kyoto, Japan).

### 2.4. Statistics

Descriptive statistics, including the arithmetic mean, median, minimum, maximum, standard deviation (SD) and variation coefficient (VC) (*i.e.*, SD/mean), were performed (Table 1). Together with the SD, the VC was used to reflect the degree of discrete distribution for different metal concentrations and to indirectly indicate their activeness in the environment. Skewness was also used to reflect different distributions of the metals. The distributions of the data were then tested for normality using the Kolmogorov–Smirnov (K–S) test. For non-normally distributed metals, the data were logarithmically transformed to obtain normal distributions. In addition, correlation coefficients were calculated to determine relationships among different metals. Descriptive statistics, normality tests on the raw and log-transformed data and correlation coefficients were all performed using SPSS Version 19.0 (SPSS Inc., Chicago, IL, USA).

### 2.5. Multivariate Analyses

Principal component analysis (PCA) and cluster analysis (CA) are the most common multivariate statistical methods used in environmental studies [36,37]. PCA is a widely used technique to reduce multivariate data dimensions and explain correlations among large numbers of observed variables by extracting a smaller number of latent factors (*i.e.*, principal components or PCs) [38]. To make the results easier to interpret, a PCA with VARIMAX normalized rotation was applied; this can maximize the variances in the loading factor across variables for each factor [39]. In this study, three of four principal factors extracted from the variables were retained with eigenvalues greater than 1.0, as determined by the Kaiser criterion [40].

Cluster analysis (CA) was performed to further classify metals of different sources on the basis of similarities between their chemical properties [39]. Hierarchical CA was used to assist inidentifying relatively homogeneous groups of variables using an algorithm that starts with each variable in a separate cluster and combines clusters until only one is left [41]. As the variables have large differences in scale, standardization was performed before computing proximities, which is achieved automatically by the hierarchical CA procedure. A dendrogram was constructed to assess the cohesiveness of the clusters formed, in which correlations among metals can be seen. The CA is complementary to the PCA. Both PCA and CA were carried out using SPSS Version 19.0.

### 2.6. Risk Assessment

Three indexes, geoaccumulation index (*Igeo*), enrichment factor (*EF*) and potential ecological risk index (*PERI*), were employed to evaluate the possible environmental risks.

#### 2.6.1. Geoaccumulation Index (*Igeo*)

The geoaccumulation index (*I*geo) was originally defined by Muller and used to quantitatively measure metal contamination in sediments [48]. This index has been successfully applied to the measurement of soil pollution [49]. The *I*geo enables the assessment of contamination by comparing recent concentrations of the metals with those from pre-industrial measurements [50]. *Igeo* can be calculated using Equation (1):

(1) $$I_{geo}={\text{log}}_{2}\left(\frac{C_{n}}{1.5B_{n}}\right)$$

where *Cn* is the concentration of the measured metal in the sample and *Bn* is the pre-industrial (geochemical background) content of this metal. In this study, soil regional background concentrations of Qinghai province have been chosen as the criterion values. The constant 1.5 compensates for possible natural fluctuations in the content of a given substance in the environment, as well as detecting very small anthropogenic influences [51,52]. Seven classes of *Igeo* represent the increasing soil contamination levels (Table 2).

#### 2.6.2. Enrichment Factor (*EF*)

*EF* is an effective tool to evaluate the magnitude of HMs and to differentiate between the HMs originating from natural provenances and those from human activities, and to assess the degree of anthropogenic influence [6,39]. This method is based on the standardization of a tested metal against a reference one. Conservative elements, such as Mn, Fe, Al, Me, Sc, Ti, or Ca are generally used as reference elements for calculation of *EF* [53,54,55,56,57,58,59,60]. Since Mn has been measured in this study, it is expected to be a conservative element and be chosen as the reference element. Previous to calculating the *EF*, a series of analyses were performed to ascertain whether Mn is a conservative element. Then the *EF* can be calculated using the following formula:

(2) $$EF=\frac{{\left(C_{E}/C_{Mn}\right)}_{sample}}{{\left(C_{E}/C_{Mn}\right)}_{background}}$$

where (*C_E_/C_Mn_*)_*sample*_ is the ratio of concentration of determined metal (*C_E_*) to that of manganese (*C_Mn_*) in the surface soil sample and (*C_E_/C_Mn_*)_*background*_ is the same ratio in reference to soil background values of Qinghai province. Five contamination categories of *EF* are shown in Table 2.

#### 2.6.3. Potential Ecological Risk Index (*PERI*)

The *PERI* was proposed by Hakanson, which integrated the concentration of HMs with toxicology, environmental effect, ecological effect, and was used to assess the contamination and ecological hazard of HMs in sedimentology [61,62]. This method represents the sensitivity of the biological community to the toxic substance and illustrates the potential ecological risk caused by the overall contamination [63]. For HMs in soil, it is also a comprehensive index to reflect their effects on the ecological environment [64]. The *PERI* is computed as:

(3) $$PERI=\sum_{i}^{m}E_{r}^{i}=\sum_{i}^{m}T_{r}^{i}\times C_{f}^{i}=\sum_{i}^{m}T_{r}^{i}\times \frac{C^{i}}{C_{n}^{i}}$$

where *E^i^_r_* is the individual potential ecological risk of the *i*th metal; *T^i^_r_* is the toxic response factor of the *i*th metal, which are defined for Mn = Zn = 1, Cr = 2, Pb = Cu = Ni = 5, As = 10 and Cd = 30 [4,61,65,66,67,68]; *C^i^_f_* is the pollution index of the *i*th metal; *C^i^* is the concentration of the examined *i*th metal in soil sample; *C^i^_n_* is the evaluation reference value of the *i*th metal, which is the soil metal background values of Qinghai province in this study. Four grades of *PERI* are defined and listed in Table 2.

To obtain the comprehensive patterns of HMs, the spatial interpolation method of IDW (inverse distance weighted) was applied, with neighboring sampling points being used for the estimation of each grid point (pixel of map). All concentration maps and assessment maps of HMs were produced by ArcGIS Version 10.1 software (Esri Inc., Redlands, CA, USA).

## 3. Results and Discussion

### 3.1. Basic Statistics and Concentration Comparisons

Descriptive statistics of the HM concentrations in surface soils of the upper reaches of the Heihe River and background values of the upper continental crust [42], world soils [43,44], Tibetan soils [35,46], soils from Qinghai and China [45] are presented in Table 1. The mean concentrations of Cr, Mn, Ni, Cu, Zn, As, Cd and Pb in the upper reaches of the Heihe River are 57.29, 818.84, 70.22, 56.38, 178.68, 21.60, 2.93 and 37.35 mg/kg, respectively. Each HM had a wide range of values with the exception of Mn. The latest Tibet soils research [35] shows the same features in variation (Table 1).

Compared with the mean metal concentrations of the upper continental crust, all eight HMs show enriched values. Compared to the mean values of world soils, slightly higher values of Ni, Cu, Zn, As and Cd are observed, while Pb levels are similar. Only Cr and Mn showed lower average concentrations than the world soil mean, rendering them distinct from the other HMs. The mean concentrations of Mn, Ni, Cu, Zn, As and Pb, but not that of Cr, are above the average values for China [45]. The mean Cd concentration is approximately 30 times higher than the average of the Chinese soils [45].

Studies of Tibetan soil background values from the 1990s to the 2010s show that the concentrations of HMs have been increasing in Tibet over the past 20 years [35,46]. For our study area, almost all HMs had higher mean values than both Tibetan soils in 2012 and the Qinghai background soils in 1990, except for the Cr. The mean concentrations of Cu and Zn were more than two times higher than those of the most recent Tibetan soils, while that of Cd was nearly 21 times higher. However, the mean concentration of Cr was less than the average of Tibetan and world soils, as well as background values of Qinghai and Chinese soils.

**Figure 2:**
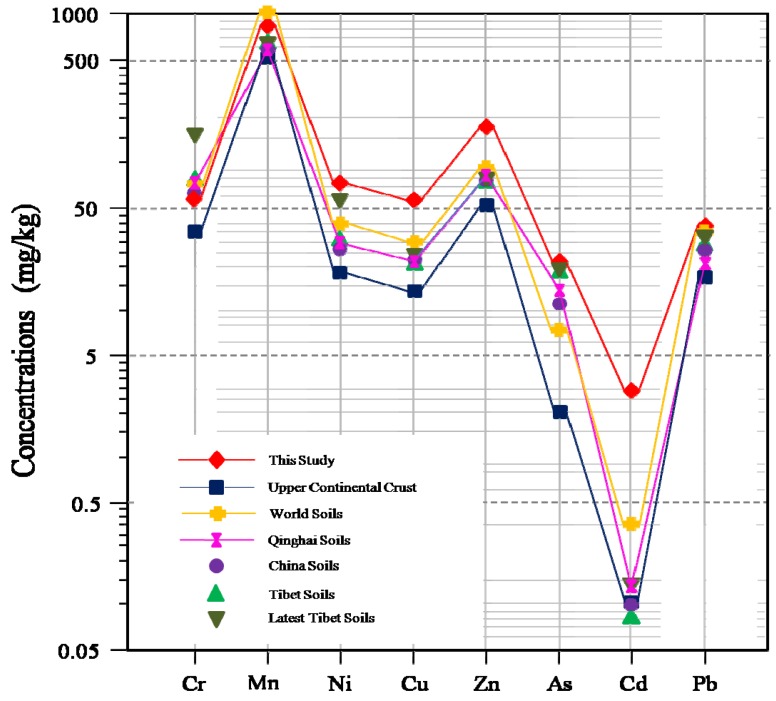
Comparisons of HM concentrations across different scales and regions.

The concentrations of HMs in this study area had the same patterns as those of previous studies in Tibet (Figure 2 and Table 1). Comparison analysis indicates that the weathering products of the underlying bedrock may be the main origin for HMs in the upper reaches of the Heihe River. Cd showed the abnormal concentration, which expands extremely high levels and warrants further statistical analysis and source identification.

### 3.2. Probability Distribution

Prior to performing multivariate analysis, it is necessary to check the probability distribution features of the variables [35]. The shape parameters and the results of K–S test for normality are presented in Table 1. Skewness values indicate that only Cd approaches a normal distribution (K-S p value = 0.809), while the other metals are positively skewed towards lower concentrations. This can be further confirmed by the median concentrations of these metals, which are much lower than the means. Metals with skewness values lower than 2, including Cr, Mn and As, still approached normal distributions, as inferred by K-S p values greater than 0.05. Kurtosis values indicate the tendency of Cr and As to be approximately normal distributed, while those of Mn and Cd are also close to normal.

The skewness and kurtosis coefficients were large for Ni, Zn, Pb and especially for Cu, showing heterogeneous concentration distributions. This may be due to the extremely high concentrations of several samples. Only two metals, Mn and Cd, passed the K-S test, with the K-S p values greater than 0.05 in the 56 original samples. We therefore performed log-transformations for the remaining HMs; however, Cr and Ni still did not pass the K-S test. Using the mean values plus three times the SDs of Cr and Ni concentration as a threshold, four outliers were found. After subtracting these four samples, log-transformed concentrations of all metals passed normality tests (Table 1). The four eliminated samples were not further used in multivariate analysis or risk assessment.

Based on VCs, the examined metals can be classified into two groups: Mn, As and Cd, whose VCs are lower than 0.5, and others whose VCs range from 0.68 to 1.33. One could expect that metals primarily from a natural source would have low VCs while the VCs of metals impacted by anthropogenic sources would be quite high. This is the case for soils or dusts, as they have undergone erosion and aeolian transport before ultimate deposition and have therefore been fully mixed [39]. The VCs of all metals were low in this study, with the highest value being 1.33 and the average VC value being 0.71 for the seven other metals. Thus, it is likely that all of the metals originate from natural sources.

### 3.3. Correlation Coefficient Analysis (CCA)

Pearson’s correlation coefficients of HMs and TOC in surface soils in the upper reaches of the Heihe River are summarized in Table 3. Cr and Ni are strongly positively correlated (0.671), which may suggest a common origin. Arsenic (As) is also somewhat associated with Cr and Ni based on their coefficients (0.441, 0.383). The correlation coefficients of paired comparisons among Cd, Pb and Zn are all above 0.4, indicating that these three metals are positively correlated. Nickel (Ni) is correlated to TOC (0.427), indicating that the content and distribution of Ni is somehow controlled or influenced by soil TOC. Manganese (Mn) and Cu are poorly correlated with the others, indicating different features; however, all metals may still originate from soils.

### 3.4. Principal Component Analysis (PCA)

**Figure 3:**
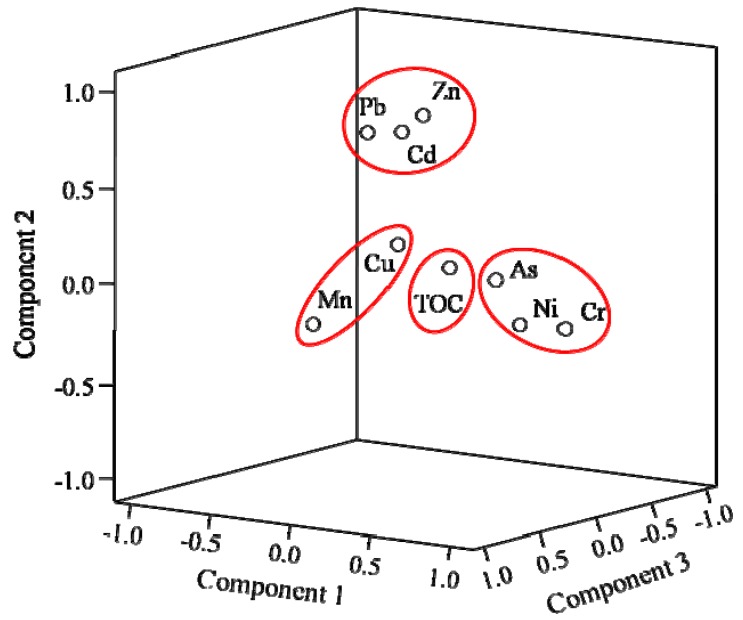
3-D PCA loading plot for 8 HMs and TOC.

After eliminating the four outliers based on the results of probability distributions, the remaining fifty-two soil data sets were subject to multivariate analysis using PCA and CA. Table 4 displays the factor loadings with a VARIMAX rotation, as well as the eigenvalues. A 3-D plot of the PCA loadings is presented in Figure 3, and the relationships among HMs and TOC can be seen. From the rotated component matrix for the PCA (Table 4), four PCs were extracted, accounting for 73.862% of the total variance. Based on the PC loadings, the eight HMs and TOC can be grouped into four PCs (F1–F4).

F1: Chromium (Cr), Ni and As are associated in this factor, displaying high loadings of 0.885, 0.755 and 0.695, respectively. Factor 1 accounts for 27.277% of the total variance. Chromium (Cr) and Ni are strongly associated, while As also shows a relatively high affiliation with these two metals. Soils developed from ultramafic rocks are usually enriched in Cr, Ni and As [35,69,70,71]. The collision of the Eurasian and Indian plates led to the uplift of the Tibetan Plateau, and the northward thrust of the Lhasa terrane likely resulted in the wide distribution of ultramafic rock throughout the Tibetan Plateau, including the upper reaches of the Heihe River [72,73,74]. Therefore, soils developed from ultramafic rocks in study area are expected to have high concentrations of Cr, Ni and As, for these metals have similar geochemical behaviors. A similar pattern of natural abundance and correlation for these metals was also found in the crust of the Earth and Tibetan soils [75].

F2: The second factor consists of Zn, Cd and Pb and accounts for 22.593% of the total variance. The loadings were 0.822, 0.816 and 0.719 for Zn, Cd and Pb, respectively. These three metals are commonly found together in various types of ore deposits [43,76]. The upper reaches of the Heihe River originates in the Qilian Mountains, which is in the Qilian metallogenic belt, one of the six grand metallogenic belts in the Tibetan Plateau. Thus, the study area is enriched in polymetals, such as Zn, Pb, Cu, Fe and Mn. Therefore, it is likely that Zn, Cd and Pb primarily share a natural source feature. This factor is taken as a natural lithogenic factor.

F3: Factor 3 is dominated by Mn and Cu and accounts for 14.239% of the total variance. However, Mn, which had a very high loading value of 0.908, is not closely related to Cu, which had a loading value of 0.574. As seen in Figure 3, Mn and Cu are separated by a long distance in the 3-D PCA loading plot, which suggests that the two metals are poorly correlated and have different features, as was observed earlier in a correlation coefficient analysis. Mn is often considered to be a conservative metal and was chosen as a reference element due to its stable physicochemical characteristics; this distinguishes it from the other HMs [39]. This factor can be treated as a conservative element factor.

F4: The fourth factor includes TOC and Ni, with loading values of 0.907 and 0.419, respectively. The high loading of TOC, which is much higher than that of Ni, indicates that TOC is the dominating contributor to this factor. This correlation indicates that the TOC concentration may, to some extent, impact the concentration of Ni in soils, especially in wet or nutrition-enriched areas [35]. This is probably due to the particular topographic feature: thermokarst. Thermokarst initiates when the ground subsides following thaw of seasonal frozen soil or permafrost [77]. After a thermokarst pond has formed, the pond size may change due to continued frozen soil thaw, variations in air temperature and precipitation [78], or drainage through open taliks, degrading ice-wedge networks, and eroding gullies [79,80]. The existence of frozen soil layer will impede the progress of vertical infiltration of water and nutrition, especially in the seasonal frozen regions [81]. Based on this feature, we conclude that soil organic matter may actively chelate some of the Ni in specific environment. F4 is a special factor of geochemical behavior.

### 3.5. Cluster Analysis (CA)

**Figure 4:**
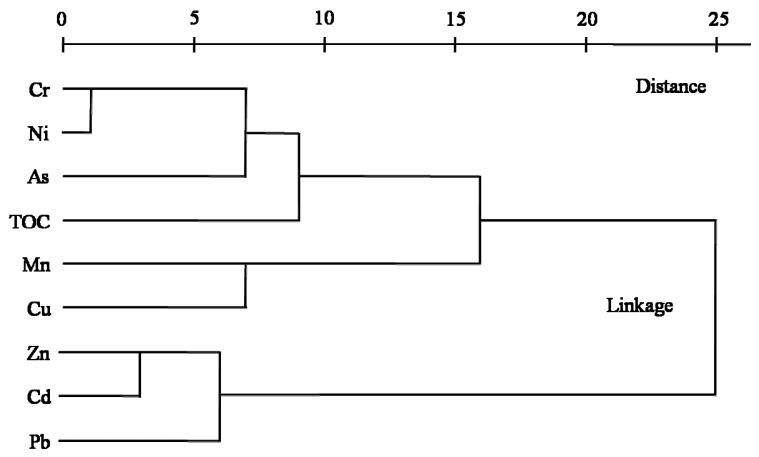
Hierarchical dendrogram of eight HMs and TOC contents.

CA was applied to the standardized bulk concentration data using Ward’s method. Euclidian distances were used as the criterion for forming clusters of HMs and TOC. A tree diagram, which was generated from Figure 4, displays four clusters: (1) Cr–Ni–As, (2) TOC, (3) Mn–Cu and (4) Zn–Cd–Pb. Clusters (1), (2), (3) and (4) reflect F1, F4, F3 and F2 in the PCA respectively, and the large distance between TOC and other clusters suggests that the TOC may have confined connections with specific metals. The four clusters have strong relations with the four PCA factors, further confirming the reliability of the PCA results and suggesting a common source from nature.

### 3.6. Spatial Distributions

**Figure 5:**
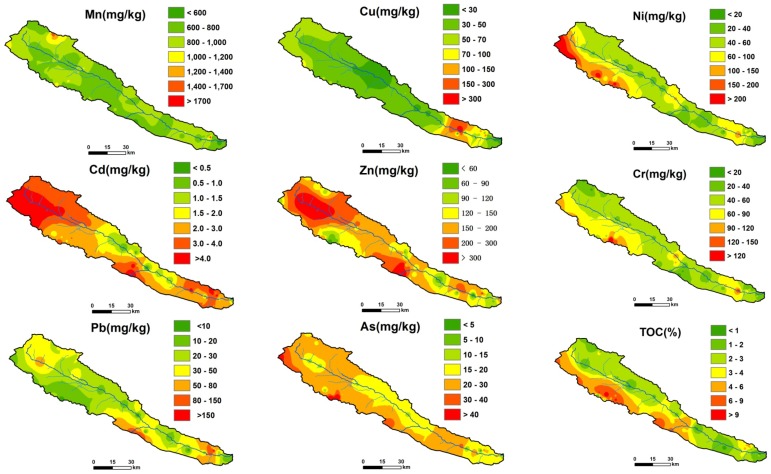
Spatial distribution of HMs and TOC in the upper reaches of the Heihe River.

The interpolated concentration maps of HMs and TOC are presented in Figure 5. Manganese (Mn) and Cu have similar distribution patterns. High levels of the two HMs, indicated by orange and red colors on the maps, were only found in a small localized area. Clastic sediments, as well as other sedimentary and metasedimentary rocks, are well distributed in the upper reaches of the Heihe River. Shales, limestones and mudstones from the bedrocks potentially contain relatively high concentrations of Mn and Cu [82,83]. These two HMs in soils should originate from the weathering of parent rocks. In other areas, the interpolated concentrations were all similar to the average world background level. Based on this analysis, these two HMs, particularly Mn, are considered to be relatively conservative elements.

Similar spatial distribution patterns were also found for Ni and Cr. The high concentration areas marked by dark red color on the map (Figure 5) shows one belt located in the southwest of study area and extending from Yinda to Yeniugou. As a prospective metallogenic region with potential for the exploitation of multiple metal ores, significant prospecting activities have been conducted in this area. There is a nickel mining facility near Yinda, which may explain the high level of Ni. Chromium (Cr) and Ni are usually associated during the mineralization processes. Therefore, it is logical that relatively high levels of Cr were also observed in this belt; this revealed that the source of these metals could be bedrock. High levels of TOC were also observed in this region where thermokarst ponds are widly spread. Elements chelated by soil organic matter are important for explaining the storage and distribution of metals [84].

For Cd, Zn, Pb and As, the hot spots covered most areas in the upper reaches of the Heihe River, especially the northwest, middle and southeast. The extremely high Cd concentration areas (in orange and dark red colors on the map in Figure 5) are distributed in the regions where the limestone is well preserved. This is probably due to cadmium carbonate (CdCO_3_) precipitation, which occurs when carbonate ions react with cadmium ions during the weathering and soil-forming processing of limestone [46,85]. The adsorption of cadmium ions by fine particles, which are substantially produced during weathering and soil-forming processes, may also contribute to high Cd concentrations. Furthermore, the drastic peeling activities of bedrocks resulting from the exploitation of mineral resources may impact the soil Cd concentration by accelerating the weathering processing.

It has been demonstrated that although Pb is highly related to soil organic matter and pH, which were not discussed in this paper, it is also positively correlated with Zn and Cd; moreover, arsenic (As) is closely correlated with Cd [46]. The same interrelationships and distributed patterns among these metals were also presented in this study (Figure 5), which further support the relationships with geological mineralization processes. This is because all of these metals (*i.e.*, Cd, Zn, Pb and As) are chalcophilic elements and share the same geochemical behaviors and properties.

Generally, Cd, Zn, Pb and As are found together in nature in several types of rocks, including ores. A variety of bedrocks, such as volcanic rocks, clastic sandstones, shales, limestones and metamorphic rocks, create the required conditions for the famous Qilian polymetallic ore deposit in the Tibetan Plateau. Soils developed on these rocks have high concentrations of Cd, Zn, Pb and As, indicating inheritance from parent rocks.

### 3.7. Environmental Risk Assessment

**Figure 6:**
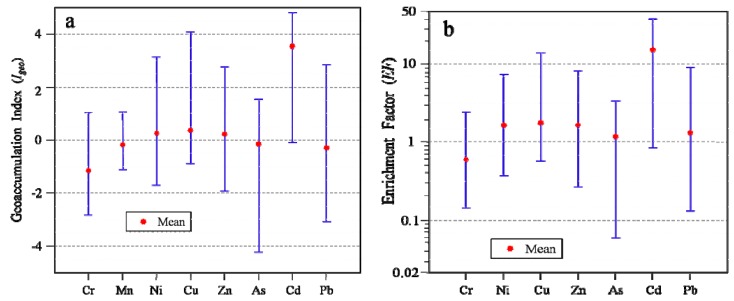
Mean, maximum and minimum values of (**a**) geoaccumulation index and (**b**) enrichment factor of HMs.

To evaluate which HM showed relatively higher risk in surface soils in the upper reaches of the Heihe River, *Igeo* and *EF* were calculated (Figure 6). The mean *Igeo* values for Cr, Mn, As and Pb are all lower than 0, indicating the absence of contamination. Meanwhile, the mean *Igeo* of Ni, Cu and Zn, are greater than 0, but smaller than 1, indicating minor contamination levels. The mean *Igeo* of Cd (3.56) is much higher than that of any other HMs, falling into the fifth category of the index (Table 2), which indicates heavy contamination in the soil. According to the CCA, PCA, CA and spatial distributions analyses, Cd in surface soils in study area is mainly derived from the underlying bedrocks, but its release is accelerated by mining activities. The high value of *Igeo* for Cd demonstrates that the soils may have a higher risk for Cd, which is due to natural origins. The mean *Igeo* values of the eight HMs decreased in the order of Cd > Cu > Ni ≈ Zn > As ≈ Mn > Pb > Cr.

Our analyses have confirmed that Mn is a conserved element in the studied environment. The *EFs* of the remaining metals were calculated and shown in Figure 6b. It is clear that the *EF* value of Cd is the largest, indicating high enrichment of the metal in soils. The mean *EF* values of the remaining six metals are all less than 2, indicating non-existent to minimal enrichment in the soil. The mean *EFs* of the metals (excluding Mn) decreased as follows: Cd > Cu ≈ Zn ≈ Ni > Pb > As > Cr. Taking both *Igeo* and *EF* calculations into account, Cd displayed an environmental risk well above those of the other metals.

**Figure 7:**
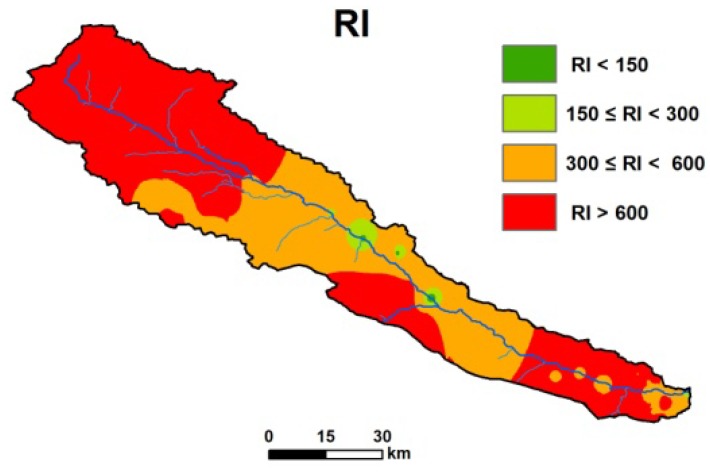
Interpolated *RI* map and spatial distribution of potential ecological risk regions.

Apart from single metal risk assessments, an *RI* calculation was also employed, to provide information about the regions with potential ecological risk in the upper reaches of the Heihe River. The interpolated *RI* map (Figure 7) displays a similar spatial distribution to that for the concentration of Zn in the northwest, middle and southeast of the study area. In other parts, Cd, whose concentration exceeds the background content to the largest extent, dominates the pattern of the *RI* map. The dark red and orange areas represent medium to high risk (*RI* value > 300) and cover almost all territory in the upper reaches of the Heihe River. The highest risk regions are located in the northwest (the entire area from Yinda to Reshui daban), the middle bottom (from Kekeer to Kekeli) and the southeast (around Zamasheng) of the study area. Fragmentary green spots only appear near Yeniugou and Dageda. The *RI* map presents integrated results after the simultaneous processing of different independent metals and yields a single result.

## 4. Conclusions

In this study, the concentrations of Mn, Ni, Cu, Zn, As, Cd and Pb have been found to be elevated in the upper reaches of the Heihe River. All seven HMs are above the background levels compared with the soils of the world, China, Tibet and Qinghai Province. Chromium (Cr) is the only metal showing lower concentrations than the reference soils, while Cd is highly elevated compared to the reference samples. The high Cd background concentration is due to well-preserved limestone, distinctive ore-forming geological conditions and the geochemical background. HMs in soils mainly originated from the weathering of underlying bedrocks. However, anthropogenic activities, such as geological exploration and mineral resources exploitation, may expedite the weathering process.

The high Cd background concentration and elevated metal content may have a long-term impact on the local population, livestock and wildlife in health, reproduction and survival, specifically in the Yinda, Reshui daban, Kekeli and Zamasheng regions. The results of this study can serve as a reference for future studies of environmental geochemistry in the upper reaches of the Heihe River to assess the impacts of Cd and HMs on biology and to evaluate the geochemical anomalies with respect to prospective ore deposits. To further clarify the background conditions and evaluate the environmental risks of HMs, additional soil samples should be collected in the areas influenced by mining activities to obtain more accurate information about changes and distributions of metal concentration in the upper reaches of the Heihe River.

## Figures and Tables

**Table 1:** Basic descriptive statistics of the HM concentrations of surface soils in the upper reaches of the Heihe River and comparisons with various studies (mg/kg; TOC:%).

Element	Raw Data									Log-Transformed Data			UCC ^a^	World Soils	China Soils ^d^	Tibet Soils ^e^		Latest Tibet Soils ^f^				Qinghai Soils ^g^		China Soil Quality Standard ^h^		
	Min	Max	Mean	Median	S.D.	V.C.	Skewness	Kurtosis	K-S p	Skewness	Kurtosis	K-S p	Mean	Mean	Mean	Mean	Median	Min	Max	Mean	Median	Mean	Median	Class 1	Class 2	Class 3
Cr	14.91	214.25	57.29	44.30	38.91	0.68	1.881	4.496	0.082	0.281	−0.223	0.949	35	70 ^b^	61.0	77.4	69.6	17.77	3429	155.54	60.06	70.1	63.3	90	350	400
Mn	413.19	1807.32	818.84	794.29	257.96	0.32	1.978	6.168	0.349	0.573	1.579	0.835	527	1000 ^b^	583	626	586	224.6	1776	617.36	591.45	580	570			
Ni	13.97	395.73	70.22	40.78	71.04	1.01	2.917	9.691	0.004	1.023	0.807	0.092	18.6	40 ^c^	26.9	32.1	29.8	6.108	2045	55.86	26.55	29.6	29.1	40	60	200
Cu	18.04	551.01	56.38	37.49	75.16	1.33	5.863	38.303	0.000	1.728	5.231	0.417	14.3	30 ^b^	22.6	21.9	19.7	6.14	107.9	24.27	22.30	22.2	22.3	35	100	400
Zn	31.87	830.06	178.68	135.81	151.31	0.85	2.726	8.493	0.002	0.494	0.925	0.481	52	90 ^b^	74.2	73.7	71.9	31.39	315	75.59	64.45	80.3	75.7	100	300	500
As	1.12	60.80	21.60	19.73	10.40	0.48	1.774	4.517	0.054	−2.369	13.053	0.156	2	7.2 ^b^	11.2	18.7	17.2	1.834	154.5	19.27	15.32	14.0	13.0	15	25	40
Cd	0.20	5.90	2.93	2.92	1.47	0.50	0.180	−0.664	0.809	−1.687	3.621	0.181	0.102	0.35 ^b^	0.097	0.08	0.074	0.028	0.849	0.141	0.108	0.137	0.132	0.2	0.6	1
Pb	3.78	225.52	37.35	22.72	41.32	1.11	2.753	8.687	0.001	0.419	0.419	0.518	17	35 ^b^	26.0	28.9	27.7	9.787	153.9	32.15	26.08	20.9	20.4	35	350	500
TOC	0.44	12.33	3.41	2.37	2.65	0.78	1.354	1.677	0.108	−0.126	−0.631	0.969														

UCC upper continental crust. ^a^ Element concentrations in the upper continental crust [42]; ^b^ Background values of the world soils [43]; ^c^ Background values of the world soils [44]; ^d^ Background values of China soils [45]; ^e^ Background values of Tibet soils [46]; ^f^ Heavy metal concentrations of Tibet soils [35]; ^g^ Soil background values of Qinghai province [45]; ^h^ China Environmental Quality Standard for Soils [47].

**Table 2:** Classification and description of geoaccumulation index (*I_geo_*), enrichment factor (*EF*) and potential ecological risk index (*PERI*).

Value	Soil Quality	Value	Enrichment Level	Value	Ecological Risk
*I_geo_* < 0	Practically uncontaminated	*EF* < 2	Deficiency to minimal enrichment	*RI* < 150	Low risk
0 < *I_geo_* < 1	Uncontaminated to moderately contaminated	2 < *EF* < 5	Moderate enrichment	150 ≤ *RI* < 300	Moderate risk
1 < *I_geo_* < 2	Moderately contaminated	5 < *EF* < 20	Significant enrichment	300 ≤ *RI* < 600	Considerable risk
2 < *I_geo_* < 3	Moderately to heavily contaminated	20 < *EF* < 40	Very high enrichment	600 ≤ *RI*	High risk
3 < *I_geo_* < 4	Heavily contaminated	40 < *EF*	Extremely high enrichment		
4 < *I_geo_* < 5	Heavily to extremely contaminated				
5 < *I_geo_*	Extremely contaminated				

**Table 3:** Pearson’s correlation matrix for HM concentrations and TOC content.

	Cr	Mn	Ni	Cu	Zn	As	Cd	Pb	TOC
Cr		0.412	0.000	0.173	0.124	0.001	0.302	0.013	0.063
Mn	0.031		0.105	0.021	0.227	0.075	0.105	0.245	0.450
Ni	**0.671**	0.177		0.208	0.065	0.003	0.335	0.045	0.001
Cu	0.134	0.283	0.115		0.286	0.046	0.007	0.274	0.296
Zn	−0.163	−0.106	−0.213	0.080		0.295	0.000	0.001	0.311
As	**0.441**	0.203	**0.383**	0.236	−0.076		0.083	0.365	0.142
Cd	−0.074	0.177	0.061	0.337	**0.555**	0.195		0.002	0.260
Pb	−0.309	−0.098	−0.237	0.085	**0.416**	−0.049	**0.400**		0.242
TOC	0.215	−0.018	**0.427**	-0.076	−0.070	0.151	0.091	0.099	

The left lower part is the correlation coefficient; the right upper part is the significance level.

**Table 4:** Rotated component matrix for 8 HM concentrations and TOC content.

Element	Rotated Component Matrix				Communities
	F1	F2	F3	F4	
Cr	0.885	−0.179	−0.094	0.061	0.827
Mn	0.015	−0.099	0.908	0.081	0.842
Ni	0.755	−0.145	0.114	0.419	0.780
Cu	0.311	0.298	0.574	−0.307	0.609
Zn	−0.058	0.822	−0.172	−0.165	0.736
As	0.695	0.107	0.239	0.010	0.552
Cd	0.136	0.816	0.292	0.040	0.771
Pb	−0.299	0.719	−0.021	0.223	0.656
TOC	0.210	0.077	-0.032	0.907	0.874
Initial Eigenvalue	2.455	2.033	1.282	0.878	
Percent of variance	27.277	22.593	14.239	9.754	
Cumulative percent	27.277	49.869	64.109	73.862	

Extraction method: Principal Component Analysis. Rotation method: Varimax with Kaiser normalization.
